# Meta-analysis of the actions of antithymocyte globulin in patients undergoing allogeneic hematopoietic cell transplantation

**DOI:** 10.18632/oncotarget.14719

**Published:** 2017-01-17

**Authors:** Jiaojiao Yuan, Renzhi Pei, Wensi Su, Junjie Cao, Ying Lu

**Affiliations:** ^1^ Medical School of Ningbo University, Ningbo, Zhejiang, P.R. China; ^2^ Department of Hematology, Yinzhou Hospital Affiliated to Medical School of Ningbo University, Ningbo, Zhejiang, P.R. China

**Keywords:** antithymocyte globulin, graft-versus-host disease, allogeneic hematopoietic cell transplantation, meta-analysis, Immunology and Microbiology Section, Immune response, Immunity

## Abstract

Graft-versus-host disease (GVHD) is a serious complication associated with allogeneic hematopoietic cell transplantation (allo-HCT). Antithymocyte globulin (ATG) is widely used prior to allo-HCT for GVHD prevention, though evidence of its efficacy remains unclear. We therefore identified nine randomized controlled trials (RCTs), enrolling 1089 patients (554 in the ATG group and 535 in the non-ATG group) to conduct a meta-analysis of the actions of ATG in allo-HCT. A relative risk or risk ratio (RR) and 95% confidence interval (CI) were calculated for each outcome. Rabbit ATG reduced overall acute (a) GVHD (RR 0.77, 95% CI 0.67-0.89, *P* = 0.0004), grade III-IV aGVHD (RR 0.53, 95% CI 0.32-0.88, *P =* 0.01), overall chronic (c) GVHD (RR 0.52, 95% CI 0.42-0.64, *P <* 0.00001) and extensive cGVHD (RR 0.28, 95% CI 0.18-0.43, *P* < 0.00001), without increased risk of relapse (RR 1.17, 95% CI 0.91-1.49, *P* = 0.23). By contrast, horse ATG did not reduce overall aGVHD (RR 1.25, 95% CI 0.88-1.79, *P* = 0.22) or cGVHD (RR 1.67, 95% CI 0.96-2.91, *P* = 0.07). ATG marginally reduced 100-day transplant related mortality (RR 0.75, 95% CI 0.56-1.00, *P* = 0.05) without compromising overall survival or increased risk of infections. Further studies are required to evaluate the optimal dosage and formulation of ATG in different conditioning regimens of transplantation with varied sources of graft and donor.

## INTRODUCTION

Rapid advances in allo-HCT, especially the development of haploidentical transplantation, have increased the possibility of successful treatment in patients with hematological malignancies [1–6]. However, 40% to 90% of recipients experience increased morbidity, mortality and decreased quality of life due to clinically significant graft-versus-host disease (GVHD), despite substantial progress in the immunobiology of hematopoietic cell allografts [7–12]. The immunosuppressive agent antithymocyte globulin (ATG) has a relatively long half-life and suppresses or kills T cells infused with the graft [13, 14]. It has been used since the 1970s to prevent severe acute and chronic GVHD (aGVHD and cGVHD, respectively) following allogeneic hematopoietic cell transplantation (allo-HCT). Several multicenter, prospective, randomized or nonrandomized studies evaluated the efficacy of ATG against GVHD [15–18]. Nevertheless, their conclusions are conflicting. A previous meta-analysis of ATG for post-transplant GVHD prophylaxis suggested that ATG use for GVHD prevention was not generally recommended, and highlighted the need for further evaluation of the impact of ATG in allo-HCT [19]. Furthermore, the risk of relapse and infections, as well as early transplant related mortality (TRM) and overall survival (OS) following the use of ATG remain unclear [20, 21]. Accordingly, we updated the randomized controlled trials (RCTs) and performed a meta-analysis to evaluate the potential benefit and risk of ATG use in allo-HCT.

## RESULTS

### Selection and characteristics of included studies

**Figure 1:**
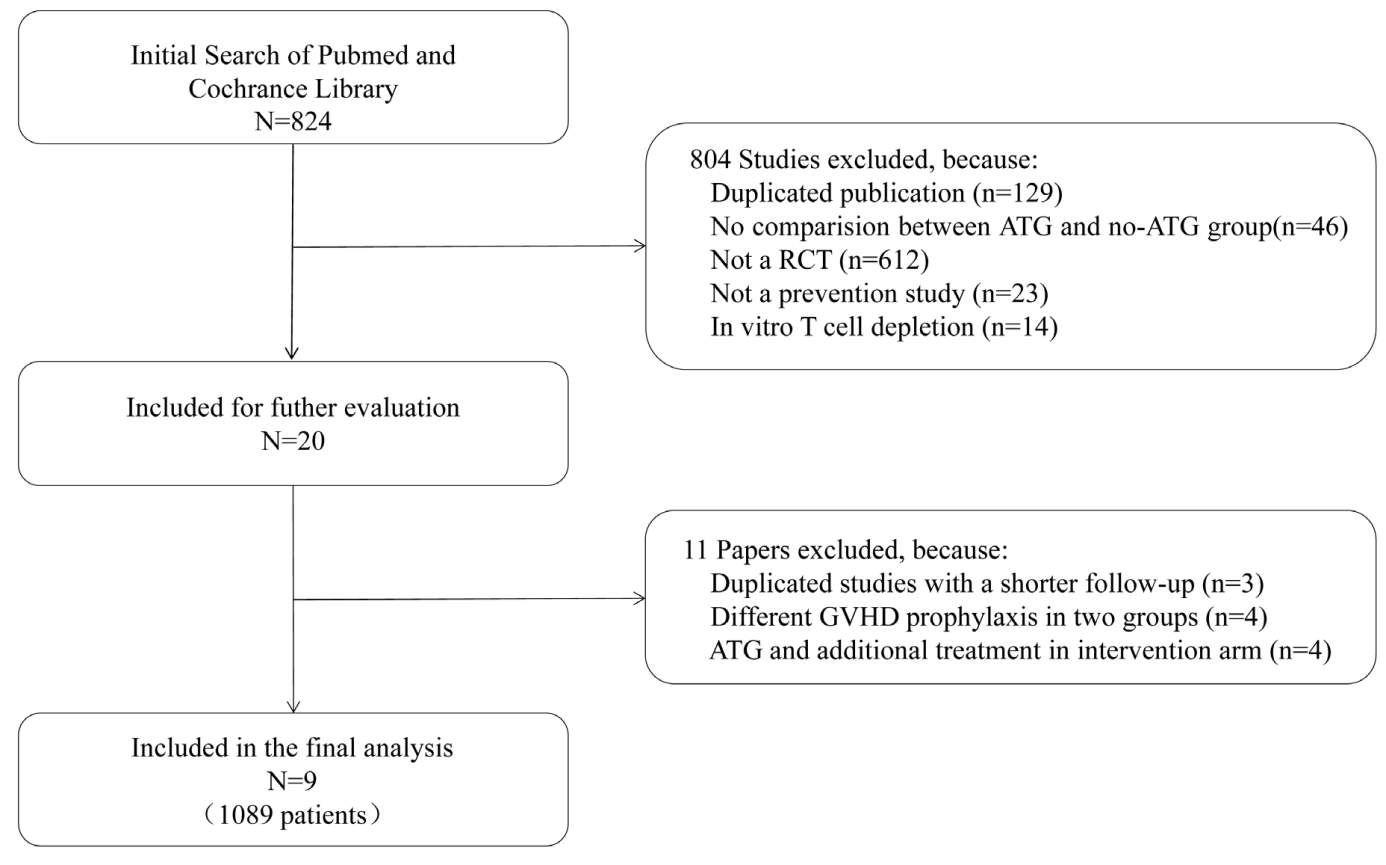
Flowchart outlining the search strategy and data selection

As illustrated in Figure 1, an online search of the PubMed and Cochrane Library databases was performed to identify and select relevant studies. We initially searched 824 studies, from which 20 were retrieved for detailed evaluation: eleven studies were eventually excluded for reasons explained in Figure 1, and nine RCTs meeting the inclusion criteria were included in the final analysis. These nine studies were published between April 1979 and January 2016, and included a total of 1089 patients: 554 in the ATG group and 535 in the control group. Patients were diagnosed with malignant hematopoietic disorders or aplastic anemia (AA). Nearly all (95%) of the patients were age 16 years or older. Six trials used rabbit ATG, either thymoglobulin (Genzyme, Cambridge, MA, USA) or ATG-Fresenius (Fresenius, Gräfelfing, Germany), and 3 trials used horse ATG. A single study used peripheral blood stem cells, five studies used bone marrow, and others used both peripheral blood stem cells and bone marrow. The median follow-up ranged from 6 to 72 months. Baseline data on the included studies are summarized in Table 1.

**Table 1:** Characteristics of included RCTs

Author	Year	ATG/Non-ATG	Disease	Age	Male sex no. (%)	Disease status at transplant no.(%)	Conditioning	Type of ATG	Dose of ATG	Graft source	Donor	Enrollment period	Median follow-up Mo (range)
Weiden, PL [26]	1979	29/27	AL+CML+LLL	17(1-32) (A) 19(2-47) (N)	16(55) (A) 19(70) (N)	CR 13(45) (A), 13(48) (N) Relapse 16(55) (A), 14(52) (N)	Cy120 mg/kg+10 GyTBI	Horse ATG	7mg/kg × 6 on alternate days post engraftment (max42 mg/kg; time to engraftment in ATG arm 14-25 days median 18)	BM	MRD	1976.6-1978.1	6~16
Donkey, KC [27]	1981	30/42	AL+CML+LLL	19 (2-53)	NR	NR	Cy120 mg/kg+10-15 GyTBI	Horse ATG	20 mg/kg × 6 alternate days from day + 7 post transplant	BM	MRD	1978.4-1979.6	>12
Bacigalupo, A [15]	2001a	29/25	Hematologic malignancies	28(18-48) (A) 29(13-51) (N)	NR	CR1/CP 18(62) (A), 15(60) (N)	Cy120 mg/kg+10-15 GyTBI	Rabbit ATG	3.75 mg/kg on days -4, -3	BM	MUD	1995.12-1997.12	28.5 (A) 34.7 (N)
Bacigalupo, A [15]	2001b	27/28	Hematologic malignancies	32(14-52) (A) 28(14-46) (N)	NR	CR1/CP 6(22) (A), 10(36) (N)	Cy120 mg/kg+10-15 Gy TBI	Rabbit ATG	3.75 mg/kg on days -5, -4, -3, -2	BM	MUD	1997.12-2000.7	17.7 (A) 18 (N)
Champlin, E [28]	2007	70/60	SAA	23(1-51) (A) 26(4-51) (N)	46 (66) (A) 35 (58) (N)	NR	Cy200 ,mg/kg	Horse ATG	30 mg/kg on days -5 to -3	98% BM	MRD	1994-2001	72
Finke, J [29]	2009	103/98	AL+CML+MDS	40 (18-60) (A) 39 (18-60) (N)	58 (56) (A) 58 (59) (N)	Early 64(62) (A), 43(43.8) (N) Advanced 39(38) (A), 55(56) (N)	Bu/Cy 120 mg/kg or Cy 120 mg/kg + TBI 8-12Gy	Rabbit ATG	20 mg/kg on days -3, -2, -1	BM or PBSC	MUD	2003.5-2007.2	48
Bacigalupo, A [30]	2010	84/86	AL+CML+other	54%>35 (A) 55%>35 (N)	Female donor male recipient 10(A), 17(N)	Early 44(52) (A), 45(52) (N) Advanced 40(48) (A), 41(48) (N)	Cy 120 mg/kg + 12 GyTBI Cy 100 mg/kg + thiotepa 10 mg/kg	Rabbit ATG	3.75 mg/kg on days -3, -2	BM or PBSC	55% MMUD 15% MMRD 30% MUD	NR	43.3(A) 48(N)
Kroger, N [16]	2016	83/72	AL	39 (18-64) (A) 43.5 (21-61) (N)	53 (63.9) (A) 40 (55.6) (N)	CR1 73 (88) (A), 66 (91.7) (N) CR2 10 (12) (N), 6 (8.3) (N)	Cy 120 mg/kg + 12 GyTBI Cy 120 mg/kg + Bu 16 mg/kg orally or 12.8 mg/kg intravenously, Etoposide 30-60 mg/kg + 12 GyTBI	Rabbit ATG	10 mg/kg on days -3, -2, -1	PBSC	MRD	2006.12-2012.2	24
Walker, I [17]	2016	99/97	AL+CL+MDS+Lympoma+other	49 (40-57) (A) 49 (40-56) (N)	63 (64) (A) 65 (67) (N)	Early 57 (58) (A), 59 (61) (N) Late 34 (34) (A), 34 (35) (N) Other 8 (8) (A), 4 (4) (N)	Myeloablative or RIC conditioning	Rabbit ATG	0.5 mg/kg on day -2, 2.0 mg/kg on day -1, 2.0 mg/kg on day +1	BM or PBSC	83% MUD 17% MMUD	2010.6-2013.8	12

NR: Not reported; AL: Acute leukemia; CL: Chronic leukemia; CML: Chronic myeloid leukemia; LLL: Lymphoblastic lymphoma with leukemia; SAA: Severe aplastic anemia; MDS: Myelodysplastic syndrome; BM: bone marrow; PBSC: peripheral blood stem cell; MRD: matched related donor; MUD: matched unrelated donor; MMUD: mismatched unrelated donor; MMRD: mismatched related donor.

### Methodological quality of RCTs

**Figure 2:**
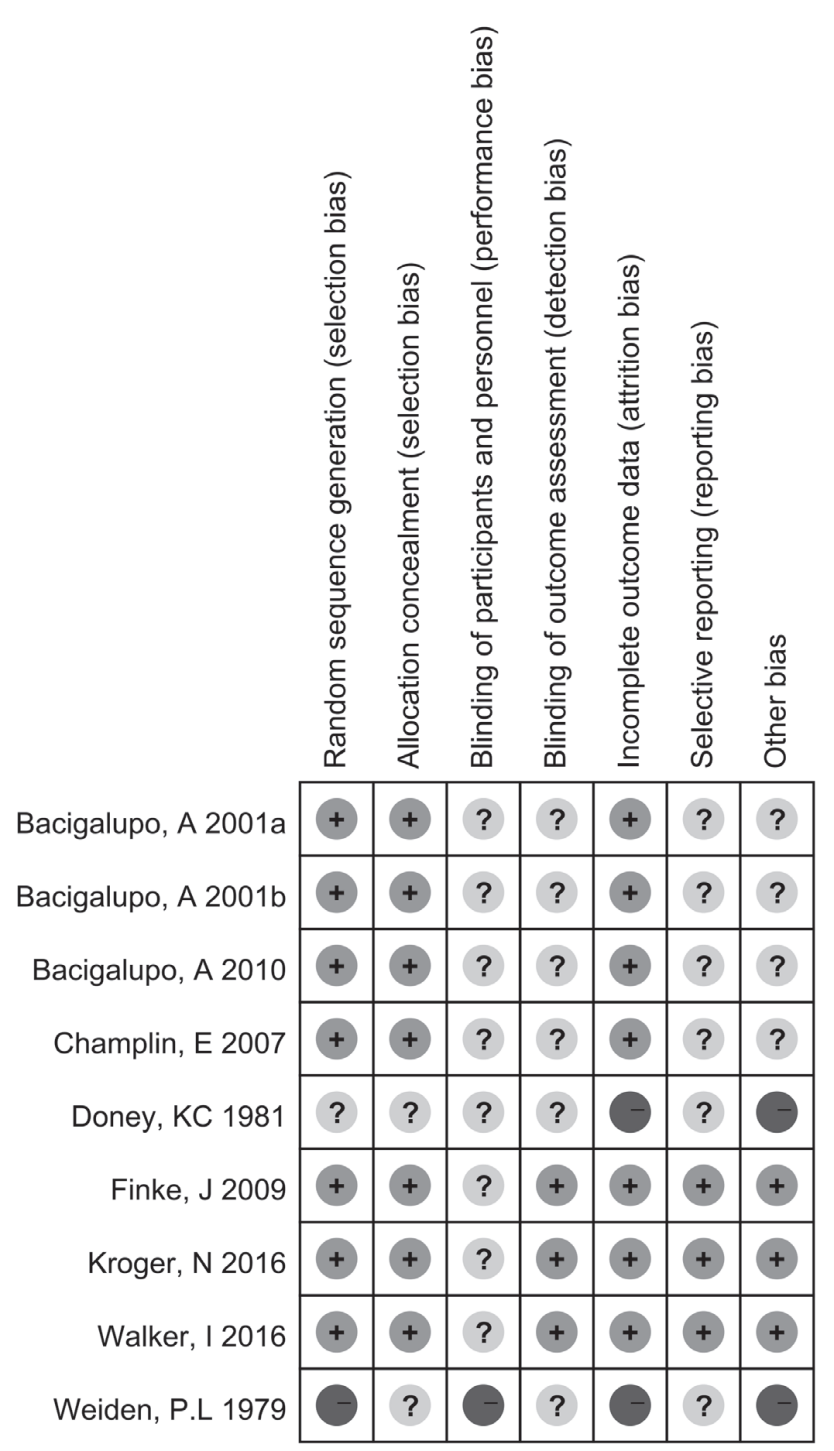
Risk of bias in the nine included RCTs

The risk bias in the included nine RCTs is shown in Figure 2. Generation of a randomization sequence, allocation concealment, and incomplete outcome data were adequately described in 78% (7/9) of RCTs. Information for the assessment of blinding was insufficient: only 33% (3/9) of RCTs were open-label studies, and none of the remaining 6 trials were described as double-blind or blinded. All of the studies described withdrawals and drop-outs. The overall risk of bias was moderate in the nine studies selected for analysis.

### GVHD

#### aGVHD (overall aGVHD and grade III-IV aGVHD)

**Figure 3:**
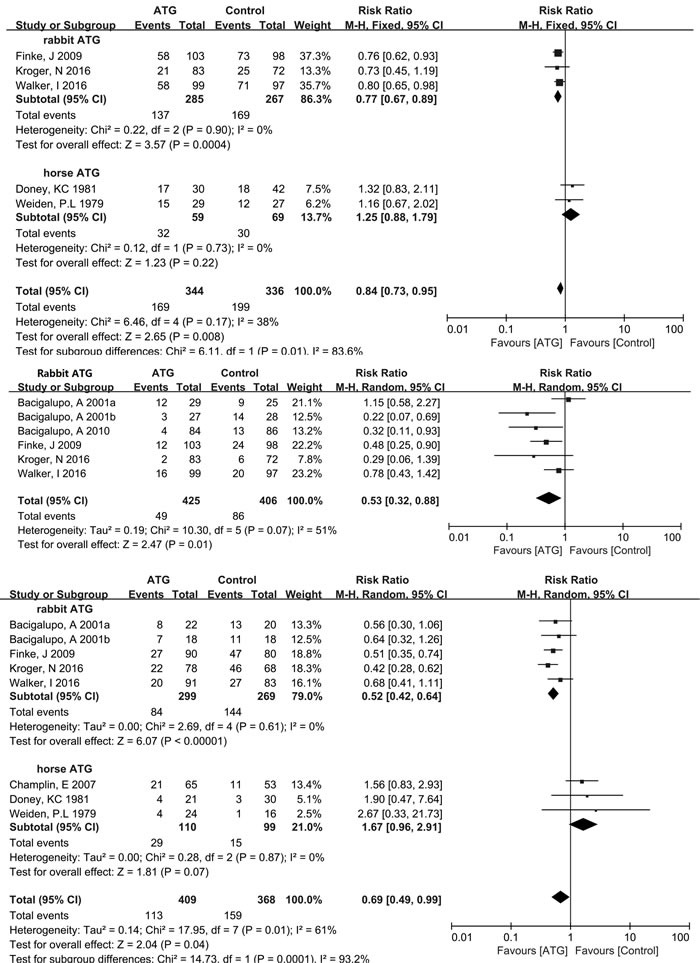

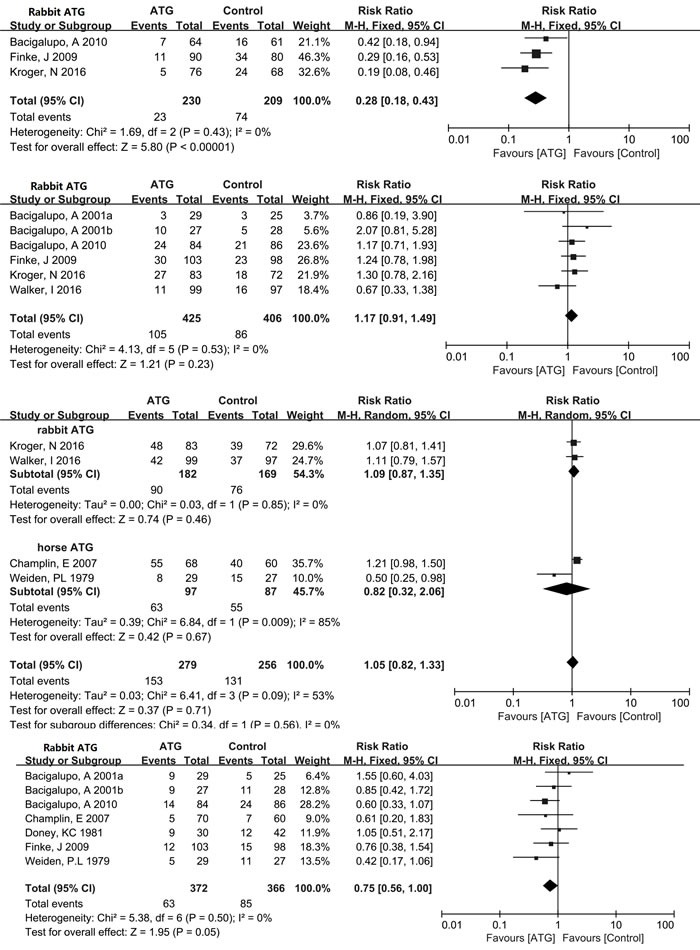

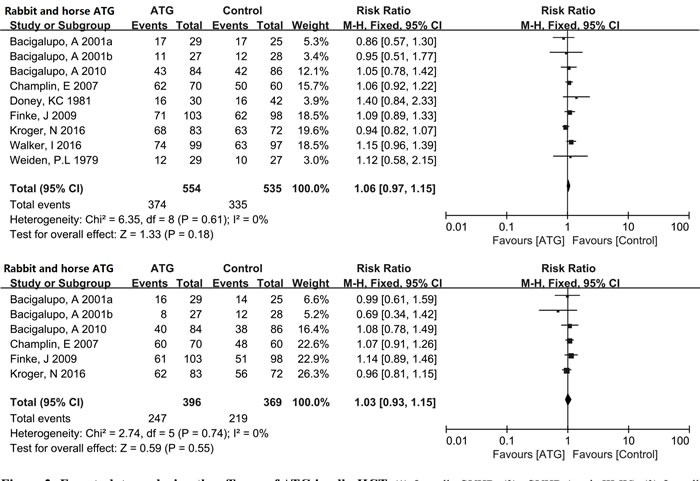
Forest plots analyzing the efficacy of ATG in allo-HCT (1) Overall aGVHD, (2) aGVHD (grade III-IV), (3) Overall cGVHD, (4) Extensive cGVHD, (5) Relapse, (6) Infections, (7) 100-day TRM, (8) 1-year OS, (9) 2-year OS.

As illustrated in Figure 3, five studies that included 680 patients were analyzed for overall aGVHD. Among the included trials, moderate heterogeneity (χ^2^ = 6.46, *df* = 4 (*P* = 0.17); *I*2 = 38%) was observed. Fortunately, in subgroup analysis, no heterogeneity (*I*2 = 0%) was found in either the rabbit or horse subgroups. Rabbit ATG effectively reduced the incidence of overall aGVHD (RR = 0.77, 95% CI = 0.67-0.89, *P* = 0.0004), whereas horse ATG was not associated with significant reduction in overall aGVHD (RR = 1.25, 95% CI = 0.88-1.79, *P* = 0.22). Six RCTs that included 831 patients treated only with rabbit ATG reported grade III-IV aGVHD data. The pooled results showed a statistically significant reduction in the rabbit ATG arm compared with the control arm (RR = 0.53, 95% CI = 0.32-0.88, *P =* 0.01). However, moderate heterogeneity (χ^2^ = 10.30, *df* = 5 (*P* = 0.07); *I*2 = 51%) was present among the included trials.

#### cGVHD (overall cGVHD and extensive cGVHD)

Eight RCTs with 777 patients were evaluated for the incidence of overall cGVHD. Statistically significant heterogeneity (χ^2^ = 17.95, *df* = 7 (*P* = 0.01); *I*2 = 61%) was observed within the pooled result. Based on subgroup analysis, rabbit ATG effectively reduced the incidence of overall cGVHD (RR = 0.52, 95% CI = 0.42-0.64, *P <* 0.00001). On the other hand, no statistical difference (RR = 1.67, 95% CI = 0.96-2.91, *P* = 0.07) in the incidence of overall cGVHD was found in the group administered horse ATG compared with the control group. And there was no heterogeneity (*I*2 = 0%) among included studies in either the rabbit or horse subgroup. Three trials comprising 439 patients used only rabbit ATG to evaluate extensive cGVHD. The pooled results showed a significant reduction in the rabbit ATG group compared with the non-ATG group (RR = 0.28, 95% CI = 0.18-0.43, *P* < 0.00001), with no heterogeneity (*I*2 = 0%) among the included RCTs.

### Relapse and infections

Six studies with a total of 831 patients all used rabbit ATG to analyze the incidence of relapse between the ATG and non-ATG arms. No heterogeneity (*I*2 = 0%) was observed among the included RCTs. The rate of relapse was similar in the rabbit ATG and non-ATG groups (RR = 1.17, 95% CI = 0.91-1.49, *P =* 0.23). Data on infections were obtained from 4 trials, with 535 enrolled patients. The pooled outcomes showed rabbit and horse ATG did not affect the incidence of infections (RR = 1.05, 95% CI = 0.82-1.33, *P =* 0.71). And moderate heterogeneity (χ^2^ = 6.41, *df* = 3 (*P* = 0.09); *I*2 = 53%) was present among the included studies.

### 100-day TRM and OS (1-year OS and 2-year OS)

Data pertaining to 100-day TRM were extracted from 78% (7/9) of the RCTs, enrolling 738 patients. A marginally significant benefit for 100-day TRM was observed in the group treated with rabbit or horse ATG for GVHD prophylaxis (RR = 0.75, 95% CI = 0.56-1.00, *P* = 0.05). There was no heterogeneity (*I*2 = 0%) among the studies. One-year OS data were extracted from all of the nine RCTs, enrolling a total of 1089 patients. And data on 2-year OS were reported by six RCTs, enrolling 765 patients. No heterogeneity (*I*2 = 0%) was observed among included studies for either 1-year or 2-year OS. The pooled hazard ratio for the comparison of the ATG arm versus non-ATG arm was RR = 1.06, 95% CI = 0.97-1.15, *P* = 0.18 for OS at 1 year and RR = 1.03, 95% CI = 0.93-1.15, *P* = 0.55 for OS at 2 years. Thus pooled outcomes showed a statistically insignificant benefit for 1-year OS and 2-year OS with the use of rabbit or horse ATG in patients receiving allo-HCT.

## DISCUSSION

GVHD is one of the most severe complications following allo-HCT [31–35]. Clinically significant GVHD (grade III-IV aGVHD and extensive cGVHD) may lead to morbidity, mortality, and poor quality of life. Unfortunately, GVHD prophylaxis with small molecule immunosuppressive drugs or pure *ex vivo*, but not *in vivo,* T cell depletion may increase the rate of relapse and infections [36–40]. Accordingly, there is an urgent need to look for a more effective therapy. ATG has been used for GVHD prophylaxis since the 1970s. However, the efficacy and risk of ATG use for prevention of GVHD are not consistent across several RCTs. We therefore conducted a meta-analysis of nine RCTs to critically evaluate the available evidence regarding the role of ATG in allo-HCT.

The final outcomes of this meta-analysis fully validated the efficacy of rabbit ATG for reducing aGVHD (overall aGVHD and grade III-IV aGVHD) and cGVHD (overall cGVHD and extensive cGVHD). By contrast, horse ATG was not associated with overall aGVHD and overall cGVHD. Unfortunately, data related to the comparative efficacy of different ATG formulations in allo-HCT are unavailable. Rabbit ATG is more efficacious than horse ATG for GVHD prevention, overall. Nonetheless, horse ATG appears more efficacious for GVHD prophylaxis in patients with aplastic anemia. Further research is required to explore the optimal treatment formulations. In addition, the immunosuppressive effect of ATG is multifactorial and not sufficiently elucidated. ATG can persist in HCT recipients for weeks to months, suppressing or killing T cells infused with the graft for a relatively long term. This is thought to be the primary mechanism by which ATG reduces GVHD [13, 14, 41–43].

It is controversial whether use of ATG increases the risk of infections and relapse after allo-HCT. Some studies suggest the potent immunosuppression achieved with ATG may delay immune recovery and thus increase the risk of infections and relapse [20, 44–47]. However, others suggest ATG only increases the risk of infections or relapse at a high dose [21, 48, 49, 50–53]. Our meta-analysis found that the rate of infectious complications was similar in the ATG and non-ATG groups, as was the rate of relapse. Why ATG does not increase the risk of infections and relapse is unknown. One possible explanation is that through opsonization and lysis after complement activation, ATG inhibits reconstitution of the T-cell pool in the peripheral blood, but it does not impair the recovery of B, NK or iNKT cells. This may enable ATG to prevent GVHD without compromising anti-pathogen defenses [54]. Furthermore, ATG may directly eliminate leukemia cells due to its broad-spectrum anti-leukemic activity, as it induces apoptosis and reduces proliferation in both leukemia cell lines and primary human leukemic cells. This is consistent with several *in vivo* studies [55–59].

Treatment with ATG resulted in a marginal reduction in the rate of 100-day TRM after allo-HCT. This is consistent with prominent aGVHD reduction. We also found that ATG is not associated with a significant benefit for 1-year and 2-year OS. Similar outcomes were reported in other randomized or non-randomized trials, with < 5-year median follow-ups [16, 60, 61]. Nevertheless, the Russell et al. study, with a 6-year median follow-up, showed marginally significant improvement of OS with 4.5 mg/kg thymoglobulin versus no ATG (*P* = 0.046) [62]. We therefore speculate the OS benefits might become apparent only during longer follow-ups.

We found significant heterogeneity among the included studies evaluating grade III-IV aGVHD and infectious complications. Further sensitivity analysis suggested data extracted from Bacigalupo et al. [30] and Weiden et al. [26] were the primary sources of heterogeneity, respectively. The sample sizes of the two studies in Bacigalupo et al. [30] were smaller (95% CI = 0.58-2.27 and 0.07-0.69, respectively) than in the other included studies, and both studies terminated patient accrual before the targeted sample size (64 patients per arm for each study) was reached. In addition, patients in the study from Weiden et al. [26] were given horse ATG as intervention, initially, but patients who developed clinically significant GVHD were randomly assigned to receive rabbit ATG. Accordingly, these were thought to be high-risk factors for introducing bias. Moreover, the included studies have other limitations reflecting differences in the duration of ATG, the graft and donor sources, conditioning regimens and primary diseases. Additional data will be necessary to comprehensively investigate the actions of ATG after allo-HCT before clinical interventions can be individualized.

## MATERIALS AND METHODS

### Inclusion and exclusion criteria

The studies included in the meta-analysis met the following criteria: i) completed original human prospective RCTs correlating ATG with GVHD after allo-HCT in hematological disorders; ii) reported data included demographics, treatment, outcome variables and sufficient information to determine the risk ratios (RRs) and 95% confidence intervals (CIs); and iii) used any dose, type and duration of ATG, myeloablative or reduced-intensity conditioning regimens, bone marrow or peripheral blood stem cells as graft sources. Exclusion criteria were: i) trials comparing different doses of ATG or different conditioning regimens; ii) retrospective surveillance analyses; and iii) editorials, comments, letters, and abstracts without full text.

### Literature search

A systematic and comprehensive search was performed independently by two authors to collect all the relevant RCTs published through July 2016 in the Medline (PubMed) and Cochrane databases. The following keywords were used to identify the available studies: antithymocyte globulin or ATG, graft-versus-host disease or GVHD, and allogeneic hematopoietic cell transplantation or allo-HCT. All the references of relevant articles were further scanned to identify studies that might be eligible for inclusion. Studies were manually selected after reading through abstracts and full texts. Any disagreement was resolved through discussion or consultation with experts in oncology and hematology. No language restrictions were used, which reduced the potential for language bias.

### Quality assessment

To minimize bias and random error, two independent reviewers evaluated the quality of the RCTs by examining the adequacy of the allocation concealment, random sequence generation, blinding of participants and personnel, blinding of outcome assessment, incomplete outcome data, selective reporting and other bias risks. The Cochrane Collaboration tool was used to assess the risk of bias for RCTs with a subjective judgment regarding protection from bias: low risk, high risk or unclear risk of bias, according to the Cochrane Handbook for Systematic Reviews of Interventions Version 5.3.0 (updated March 2011); available from http://handbook.cochrane.org/

### Data extraction and outcomes

All data regarding baseline characteristics, transplant characteristics, and outcomes were extracted. All the end points we observed were evaluated in univariate analyses. Data extraction was conducted independently by two investigators using standardized extraction of data on the benefits and harms associated with treatment (i.e., ATG versus no ATG). Any discrepancies were resolved by seeking advice from experts in hematology or statistics. The primary outcomes of interest were efficacy as measured by the incidence of aGVHD (overall aGVHD and grade III-IV aGVHD) and cGVHD (overall cGVHD and extensive cGVHD). Secondary outcomes included the incidence of relapse, infections, 100-day TRM and OS at 1 year and 2 years.

### Data analysis and statistical methods

For each trial, binary outcomes were calculated as RRs with 95% CIs. The power of each study was calculated using the Power and Sample Size Calculation program [22]. Statistical heterogeneity across the studies included in the meta-analysis was assessed using Cochrane's Q statistic and χ^2^ test with a significance level of *P* < 0.1 to select the type of analysis [23]. The fixed-effects model was used for the analysis with an *I*2 < 50%, whereas the random-effects model was used for the analysis with an *I*2 > 50% [24]. The *I*^2^ statistic was calculated to quantify possible heterogeneity: *I*^2^ > 30% moderate heterogeneity, *I*^2^ > 75% considerable heterogeneity. We conducted subgroup analysis or sensitivity analysis to find out sources of heterogeneity when *I*2 > 50%. The Cochrane statistical program Review Manager (Review Manager, Version 5.3) [25] was used for all statistical analyses.
